# Linker DNA and histone contributions in nucleosome binding by p53

**DOI:** 10.1093/jb/mvaa081

**Published:** 2020-07-23

**Authors:** Masahiro Nishimura, Yasuhiro Arimura, Kayo Nozawa, Hitoshi Kurumizaka

**Affiliations:** 1 Laboratory of Chromatin Structure and Function, Institute for Quantitative Biosciences; 2 Department of Biological Sciences, Graduate School of Science, The University of Tokyo, 1-1-1 Yayoi, Bunkyo-ku, Tokyo 113-0032, Japan

**Keywords:** chromatin, histone, nucleosome, p53, pioneer transcription factor

## Abstract

The tumour suppressor protein p53 regulates various genes involved in cell-cycle arrest, apoptosis and DNA repair in response to cellular stress, and apparently functions as a pioneer transcription factor. The pioneer transcription factors can bind nucleosomal DNA, where many transcription factors are largely restricted. However, the mechanisms by which p53 recognizes the nucleosomal DNA are poorly understood. In the present study, we found that p53 requires linker DNAs for the efficient formation of p53-nucleosome complexes. p53 forms an additional specific complex with the nucleosome, when the p53 binding sequence is located around the entry/exit region of the nucleosomal DNA. We also showed that p53 directly binds to the histone H3-H4 complex via its N-terminal 1–93 amino acid region. These results shed light on the mechanism of nucleosome recognition by p53.

## Graphical Abstract

**Figure:**
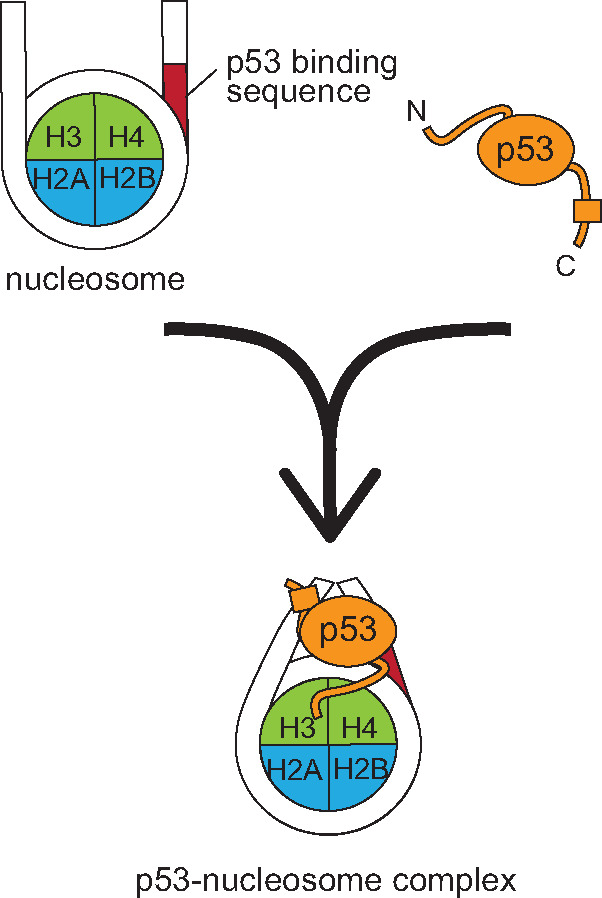

In genomes, transcription factors bind to specific DNA sequences on regulatory elements to control their target gene expression. In eukaryotes, genomic DNA is tightly compacted as chromatin, in which the repeating unit is the nucleosome (1). The nucleosome consists of the histone octamer containing two copies each of the core histones, H2A, H2B, H3 and H4 and approximately 150 bp of DNA (2). In the nucleosome structure, the backbone moiety of the DNA tightly interacts with the core histone surfaces at 10 bp intervals (3, 4). Nucleosomes are intrinsically inhibitory for DNA-binding proteins, such as transcription factors. In fact, many transcription factors apparently do not bind to their target DNA sequences within the nucleosome, but instead bind to the nucleosome-free DNA regions (5, 6). On the other hand, pioneer transcription factors, which are involved in many biological processes such as cell-fate decisions and reprogramming, recognize their target DNA sequences within the nucleosome and facilitate the transcription initiation process (7).

The tumour suppressor p53 is a pioneer transcription factor (8–11), and regulates various genes involved in cell-cycle arrest, apoptosis and DNA repair in response to cellular stress (12). The human p53 protein has three distinct functional amino acid regions (13, 14): the N-terminal region (amino acid residues 1–93) that interacts with various transcriptional cofactors, the DNA-binding region (amino acid residues 94–293) that recognizes the p53 binding sequence (p53BS) and the C-terminal region (amino acid residues 294–393) that forms a homo-tetramer (the tetramerization domain: amino acid residues 325–356) and binds to DNA independently of the DNA sequence (the C-terminal disordered region: amino acid residues 357–393). Especially, the p53 DNA-binding domain is frequently mutated in cancer cells (15).

Genomic studies have suggested that the p53 binding sites are located near the entry/exit sites of nucleosomes in cells (10, 16). In addition to the specific DNA binding by p53, non-specific DNA binding is also observed (17–19). A fluorescence recovery after photobleaching experiment revealed that the p53 mobility is extremely slow in the nucleus (20). This suggests that p53 tightly associates with chromatin in cells. The amount of the p53 protein in damaged cells is substantially increased, up to micromolar amounts (21), suggesting that the excess p53 associates with chromatin in a DNA sequence independent manner. Consistent with this idea, p53 also possesses nucleosome-binding activity independent of the DNA sequence (16). Therefore, the DNA sequence independent binding to chromatin may be important to tether p53 to chromatin in cells.

To reveal the mechanisms by which p53 binds to the nucleosomes with and without its target sequence, p53BS, we prepared the recombinant human p53 protein and performed biochemical analyses with reconstituted nucleosomes.

## Materials and Methods

### Preparation of human p53

The DNA fragments encoding the human p53 full-length (p53FL), p53 N-terminal region (1–93 amino acid residues, p53NTR) and N-terminus deleted p53 (94–393 amino acid residues, p53ΔN) proteins were inserted into the pGEX-6P-1 vector (GE Healthcare). *Escherichia coli* BL21(DE3) cells with the p53 expression plasmids were grown at 37°C until the optical density reached 1.2 (OD_600_). The expression of the GST-fused p53 proteins was then induced by the addition of 0.5 mM isopropyl β-d-1-thiogalactopyranoside, and the cells were further cultured at 18°C overnight. The cells were harvested by centrifugation and resuspended in buffer A (50 mM Tris–HCl (pH 7.5), 0.5 M NaCl, 10% glycerol, 1 mM EDTA and 2 mM 2-mercaptoethanol). The cells were then disrupted by sonication, and the lysates were centrifuged. The supernatants were gently mixed with glutathione sepharose 4B beads (GE Healthcare) at 4°C for 1 h. The beads were then loaded into an Econo-column (Bio-Rad), and were washed with 20 column volumes (CVs) of buffer B (50 mM Tris–HCl (pH 7.5), 1 M NaCl, 1 mM EDTA and 2 mM 2-mercaptoethanol), followed by washing with 20 CVs of buffer C (30 mM Tris–HCl (pH 7.5), 0.15 M NaCl and 2 mM 2-mercaptoethanol). For the electrophoretic mobility shift assay (EMSA) experiments, the GST-tags on the p53 proteins were removed by on-column GST-tagged human rhinovirus 3C protease cleavage, at 4°C overnight. The eluted proteins were further purified by Heparin Sepharose (GE Healthcare) chromatography, using stepwise elution with buffer D (30 mM Tris–HCl (pH 7.5), 1 M NaCl and 2 mM 2-mercaptoethanol), and finally purified by chromatography on a HiLoad 16/600 Superdex200 pg (GE Healthcare) or HiLoad 26/600 Superdex75 pg (GE Healthcare) column with buffer E (30 mM Tris–HCl (pH 7.5), 0.5 M NaCl and 2 mM 2-mercaptoethanol). For the pull-down assay, the GST-fused p53 proteins on the Glutathione Sepharose 4B beads were eluted with buffer F (30 mM Tris–HCl (pH 9.0), 0.15 M NaCl, 2 mM 2-mercaptethanol, 20 mM glutathione), and were finally purified by chromatography on a HiLoad 16/600 Superdex200 pg column (GE Healthcare), eluted with buffer E.

### Purification of DNA fragments

The 193 bp DNA fragments derived from the Widom 601 sequence were prepared as described (22, 23). According to a previous study (16), the original 193 bp 601 sequence contains part of the p53BS (CATG) near the entry/exit sites of the nucleosome. Therefore, the CATG base pairs were replaced with AGGT in this study. As for the 193 bp DNA with p53BS, GGGCATGTCCGGGCATGTCC (24) was inserted at the position corresponding to the entry/exit site of the nucleosome. The 24 bp oligonucleotide was prepared by annealing. All DNA sequences are listed in Table I.

**Table I.** DNA sequences

DNA fragment	Sequence
145 bp DNA	5′-ATCAGAATCCCGGTGCCGAGGCCGCTCAATTGGTCGTAGACAGCTCTAGCACCGCTTAAACGCACGTACGCGCTGTCCCCCGCGTTTTAACCGCCAAGGGGATTACTCCCTAGTCTCCAGGCACGTGTCAGATATATACATCGAT -3′
193 bp DNA	5′- ATCGGACCCTATCGCGAGCCAGGCCTGAGAATCCGGTGCCGAGGCCGCTCAATTGGTCGTAGACAGCTCTAGCACCGCTTAAACGCACGTACGCGCTGTCCCCCGCGTTTTAACCGCCAAGGGGATTACTCCCTAGTCTCCAGGCACGTGTCAGATATATACATCCAGGCAGGTTGTCGCGAAATTCATAGAT -3′
193 bp DNA with p53BS	5′- ATCGGACCCTATCGCGAGCCAGGCCTGAGAATCCGGTGCCGAGGCCGCTCAATTGGTCGTAGACAGCTCTAGCACCGCTTAAACGCACGTACGCGCTGTCCCCCGCGTTTTAACCGCCAAGGGGATTACTCCCTAGTCTCCAGGCACGTGTCAGATATAGGGCATGTCCGGGCATGTCCCGAAATTCATAGAT -3′
24 bp oligonucleotide	5′-CAGGTTGTCGCGAAATTCATAGAT -3′

### Preparation of human histones, histone complexes and nucleosomes

The preparation methods for the recombinant human histone complexes and the nucleosomes were described in Kujirai *et al.* (25). The histone complexes were refolded by dialysis, and then further purified by gel filtration chromatography. The nucleosomes were prepared with the DNA fragment and the histone octamer by the salt dialysis method. The reconstituted nucleosomes were purified by non-denaturing polyacrylamide gel electrophoresis (PAGE) with a Prep Cell apparatus (Bio-Rad).

### Electrophoretic mobility shift assay for p53 binding to the nucleosome

The nucleosomes (0.1 µM) were mixed with p53 or a p53 mutant (0–0.8 µM) in reaction solution (22 mM Tris–HCl (pH 7.5), 100 mM NaCl, 0.4 mM 2-mercaptoethanol and 0.8 mM DTT), and the samples were incubated at 25°C for 30 min. The p53 binding was visualized by non-denaturing 5% PAGE in 0.5 × TBE buffer (45 mM Tris, 45 mM boric acid, 1 mM EDTA), followed by ethidium bromide staining.

### 2D protein gel analysis of the p53-nucleosome complex

For the preparation of the p53-nucleosome complex, p53 (270 pmol) and the nucleosome (60 pmol) were incubated in reaction solution (25 mM Tris–HCl (pH 7.5), 250 mM NaCl, 1 mM 2-mercaptoethanol and 0.5 mM DTT) at 25°C for 30 min. The p53-nucleosome complex was fractionated on a non-denaturing 5–12% SuperSep™ Ace gel (FUJIFILM Wako Pure Chemical Corporation) in 1 × TBE buffer (90 mM Tris, 90 mM boric acid, 2 mM EDTA), followed by ethidium bromide staining (first dimension). The gel was soaked in denaturing buffer (2% SDS, 10% glycerol, 50 mM Tris–HCl (pH 6.8) and 100 mM DTT), and was gently agitated at room temperature for 1 h. The gel was then analysed by 18% SDS-PAGE, followed by Oriole (Bio-Rad) staining (second dimension).

### Electrophoretic mobility shift assay for p53 competitive binding to the nucleosome

p53 (0.8 µM) was incubated with the nucleosome (0.1 µM) in 10 µl of reaction solution (20 mM Tris–HCl (pH 7.5), 100 mM NaCl, 0.4 mM 2-mercaptoethanol and 0.8 mM DTT), at 25°C for 30 min. The 24 bp DNA (0–1.6 µM) was then added to the reaction solution, and incubated at 25°C for 30 min. The incubated samples were fractionated by non-denaturing biphasic PAGE, using a lower 12% acrylamide gel and an upper 5% acrylamide gel. The PAGE was performed in 0.5 × TBE, followed by ethidium bromide staining.

### Electrophoretic mobility shift assay for p53-nucleosome complex binding to PL2-6

The nucleosome acidic patch antibody, PL2-6, was prepared as described previously (*26*). p53 (0.8 µM) was incubated with the nucleosome (0.1 µM) in 10 µl of reaction solution (20 mM Tris–HCl (pH 7.5), 100 mM NaCl, 0.4 mM 2-mercaptoethanol and 0.8 mM DTT), at 25°C for 30 min. PL2-6 (0.3 µM) was then added to the reacted samples and incubated on ice for 10 min. The incubated samples were analysed by electrophoresis on a non-denaturing 5–12% SuperSep™ Ace gel (FUJIFILM Wako Pure Chemical Corporation) in 1 × TBE buffer, followed by ethidium bromide staining.

### Electrophoretic mobility shift assay for p53 binding to the histone complex

The H3-H4 complex (15 µM as dimer) was mixed with increasing amounts (0–18 µM) of p53 in 10 µl of reaction solution (30 mM Tris–HCl (pH 7.5), 150 mM NaCl, 0.1% NP-40 and 1 mM 2-mercaptoethanol), and incubated at 25°C for 30 min. The bands were visualized by non-denaturing 5% PAGE in 0.5 × TBE buffer, followed by Coomassie Brilliant Blue (CBB) staining as described (27).

### Pull-down assay for p53 binding to the histone complex

The H3-H4 complex (2.5 µM as dimer) was mixed with 2.5 µM of GST, GST-p53, GST-p53NTD or GST-p53ΔN in 20 µl of reaction solution (20 mM Tris–HCl (pH 8.0), 150–164 mM NaCl and 0.1% NP-40), and incubated at 25°C for 30 min. Glutathione Sepharose 4B beads (GE Healthcare) and 500 µl of binding solution (20 mM Tris–HCl (pH 8.0), 150 mM NaCl, 10 µg/ml BSA and 0.1% NP-40) were added to the reaction mixture, and the samples were rotated at 4°C for 30 min. The beads were collected by centrifugation, and washed three times with 1 ml of wash solution (20 mM Tris–HCl (pH 8.0), 150 mM NaCl and 0.1% NP-40). The washed beads were mixed with 10 µl of sample buffer (100 mM Tris–HCl (pH 6.8), 4% SDS, 20% glycerol, 200 mM 2-mercaptoethanol and 0.2% bromophenol blue), and were heated at 95°C for 15 min. The samples were analysed by 18% SDS-PAGE, followed by CBB staining.

## Results and Discussion

### p53 efficiently binds to the nucleosome with linker DNAs

**Fig. 1.**
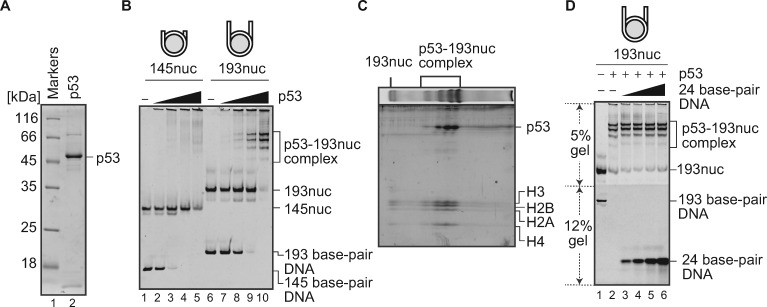
**p53 binding to the nucleosome**. (**A**) SDS-PAGE of the purified p53FL protein. Lanes 1–2 indicate protein markers and the purified p53 (800 ng), respectively. The samples were analysed by 16% SDS-PAGE, followed by CBB staining. (**B**) EMSA for p53 binding to the nucleosomes containing the 145 bp DNA (145nuc) and 193 bp DNA (193nuc). Increasing amounts of p53 (0, 0.2, 0.4, 0.6 and 0.8 µM) were mixed with 0.1 µM of 145nuc (lanes 1–5) or 0.1 µM of 193nuc (lanes 6–10). p53 binding was detected by non-denaturing PAGE, followed by ethidium bromide staining. The reproducibility was confirmed by repeated experiments. (**C**) 2D protein gel analysis of the p53-193nuc complexes. The p53-193nuc complex was fractionated on a 5–12% gradient gel, followed by ethidium bromide staining (first dimension). The gel was then analysed by SDS-PAGE, followed by Oriole staining (second dimension). The reproducibility was confirmed by repeated experiments. (**D**) Competitive nucleosome-binding assay for p53. The 193nuc (0.1 µM, lane 1) was mixed with p53 (0.8 µM). Increasing amounts of 24 bp DNA fragments (0, 0.1, 0.2, 0.4, 0.8 and 1.6 µM) were then mixed with the p53-nucleosome complex (lanes 2–7). The remaining complexes were analysed by non-denaturing biphasic PAGE: upper gel (5%) and lower gel (12%), followed by ethidium bromide staining. The reproducibility was confirmed by repeated experiments.

We first tested the nucleosome-binding activity of purified p53 (Fig. 1A). To do so, nucleosomes with and without linker DNAs were prepared with a 193 bp DNA and a 145 bp DNA derived from the Widom 601 sequence, respectively (16, 28). These DNA substrates did not contain the p53 target sequence. Our EMSA revealed that p53 formed complexes with the nucleosome containing the 193 bp DNA (with 24 bp linker DNAs), as four discrete bands on the non-denaturing polyacrylamide gel (Fig. 1B, lanes 6–10). These discrete bands may reflect p53 binding to a specific site of the nucleosome, in the absence of its target sequence. The 2D protein gel analysis revealed that these four bands appropriately contained p53 and histones, indicating that they corresponded to the p53-nuclesome complexes (Fig. 1C). Interestingly, discrete bands corresponding to the specific p53 binding to the nucleosome were not observed in the experiments with the nucleosome without linker DNAs (Fig. 1B, lanes 1–5). Therefore, p53 specifically binds to the nucleosome, even in the absence of its target DNA sequence, if the nucleosome contains linker DNAs. This is consistent with the results of a molecular dynamics simulation, in which p53 preferentially bound to the linker DNA in the nucleosome (29). p53 is known to form a homo-tetramer in the complex with DNA (30); hence, the multiple bands of the p53-nucleosome complexes may be attributed to p53 oligomerization states.

To eliminate the possibility that p53 binds to the 24 bp linker DNA regions, we next performed a competitive nucleosome-binding assay. In this assay, the nucleosome containing the 193 bp DNA was used. The competitor was a 24 bp double-stranded oligonucleotide containing the same DNA sequence as the linker DNA of the nucleosome. As shown in Fig. 1D, the p53-nucleosome complexes remained intact when the 24 bp oligonucleotide was titrated, even in the presence of a 16-fold amount of the 24 bp oligonucleotide. These results suggested that p53 does not simply bind to the linker DNAs, but specifically binds to the nucleosome with linker DNAs.

Previous single molecule studies reported that the sequence independent DNA binding of p53 may facilitate the efficient search for its target sequence in genomes (17–19). The sequence independent nucleosome binding reported here may play an important role in the target sequence search process by p53.

### p53 recognizes its target DNA sequence located around the entry/exit site of the nucleosome

**Fig. 2.**
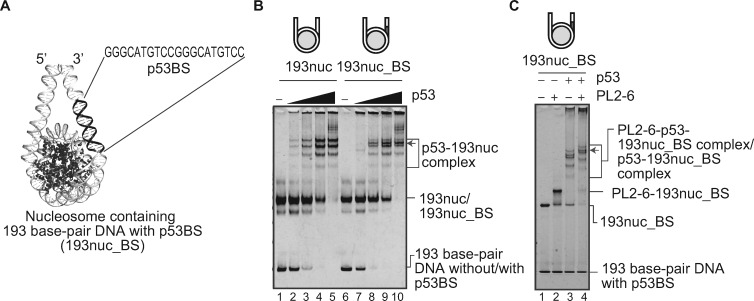
**The p53 target sequence induces specific p53-nucleosme complex formation**. (**A**) The structural representation of the predicted p53 target sequence (p53BS) location in the nucleosome containing the 193 bp DNA (193nuc_BS) (modified from PDB ID: 5NL0). (**B**) EMSA for p53 binding to nucleosomes with or without p53BS. Increasing amounts (0, 0.2, 0.4, 0.6 and 0.8 µM) of p53 were mixed with 193nuc (lanes 1–5) or 193nuc_BS (lanes 6–10). The nucleosome concentration was 0.1 µM. p53 binding was detected by non-denaturing PAGE, followed by ethidium bromide staining. The additional band is indicated by an arrow. The reproducibility was confirmed by repeated experiments. (**C**) EMSA for p53-nucleosome complex binding to the PL2-6 antibody. p53 (0.8 µM) and 193nuc_BS (0.1 µM) were mixed, and then the PL2-6 antibody (0.3 µM) was added. The samples were analysed by EMSA with a 5–12% gradient gel, followed by ethidium bromide staining. The reproducibility was confirmed by repeated experiments. The shifted additional band is indicated by an arrow.

We next tested if the p53 target sequence, p53BS, affects the p53-nucleosome complex formation by EMSA. It has been reported that p53 stably binds to the p53BS located at the entry/exit site of the nucleosome in its natural target locus *in vivo* (10). Yu et al. (16) also showed that the nucleosome containing the p53 target sequence at entry/exit sites is efficiently bound by p53, as compared to the other positions in the nucleosome. Therefore, we prepared a 193 bp Widom 601-derived DNA, in which the p53BS is inserted at the position corresponding to the nucleosomal entry/exit site, and reconstituted the nucleosome (Fig. 2A). As shown in Fig. 2B, the insertion of the p53BS did not significantly affect the nucleosome-binding efficiency of p53. However, an additional band was observed in the presence of p53, when the p53BS DNA was inserted at an entry/exit site of the nucleosome (lanes 6–10). To clarify whether the additional band consists of the p53-nucleosome complex or the p53-DNA complex, we performed EMSA in the presence of a nucleosomal acidic patch antibody, PL2-6 (26), which could induce a super shift of the nucleosome band. As a result, the additional band was shifted up by binding to the PL2-6 antibody, together with the other nucleosome bands (Fig. 2C). These results indicated that p53 recognizes its target DNA sequence at the entry/exit site of the nucleosome, and forms a specific complex. A previous nucleosome-binding study revealed that p53 binding to the nucleosomal target site is substantially suppressed, when the p53 target site is moved toward the inside of the nucleosome (10). Therefore, the nucleosomal position of the p53 target sequence may be involved in the regulatory mechanism for the p53-mediated gene expression.

The p53 binding to its target sequence in the nucleosome is probably mediated by its central DNA-binding region. However, the C-terminal region of p53 also possesses DNA-binding activity (17–19). Therefore, the central and C-terminal DNA-binding regions may cooperatively function in the nucleosomal DNA binding.

### p53 directly binds to the histone H3-H4 complex

**Fig. 3.**
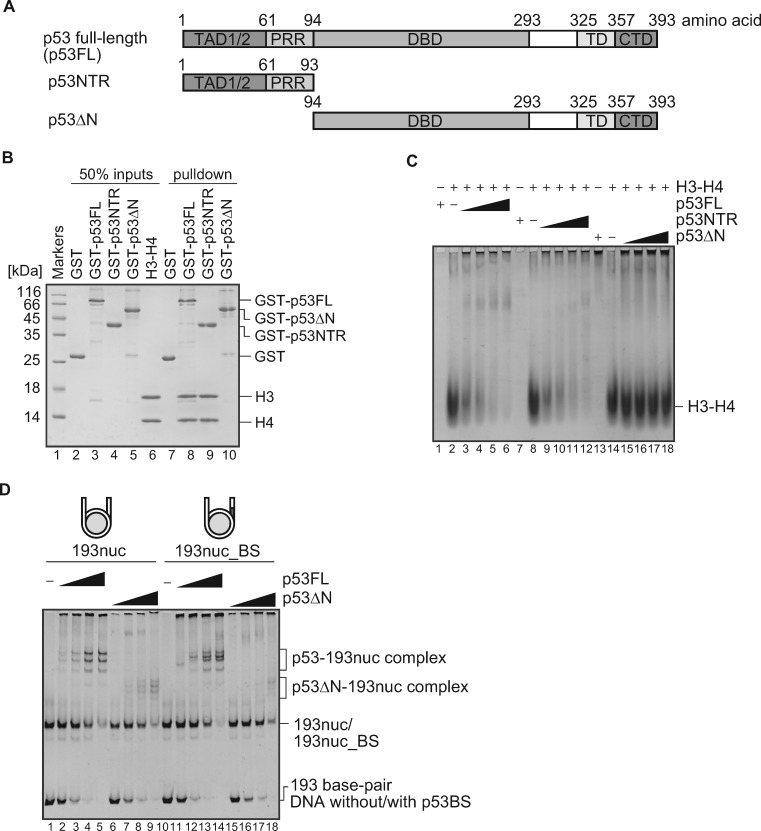
**Histone binding activity of p53**. (**A**) Schematic representation of human p53FL and its deletion mutants. Transcription activation domain 1/2 (TAD1/2), proline-rich region (PRR), DNA-binding domain (DBD), tetramerization domain (TD) and C-terminus domain (CTD) are indicated. p53NTR (amino acid residues 1–93) and p53ΔN (amino acid residues 94–393) are also presented. (**B**) Pull-down assay for p53 binding to the histone complex. Lane 1 indicates protein markers. Lanes 2–6 represent the inputs of GST, GST-p53FL, GST-p53NTD, GST-p53ΔN and the H3-H4 complex, respectively. The H3-H4 complex (2.5 µM as dimer) was mixed with 2.5 µM of GST (lane 7), GST-p53FL (lane 8), GST-p53NTD (lane 9) or GST-p53ΔN (lane 10), and was incubated with glutathione sepharose 4B beads. The proteins captured by the beads were analysed by SDS-PAGE with CBB staining. The reproducibility was confirmed by repeated experiments. (**C**) Protein EMSA for histone binding. Increasing amounts (0, 9, 12, 15 and 18 µM) of p53FL (lanes 2–6), p53NTR (lanes 8–12) or p53ΔN (lanes 13–18) were mixed with the H3-H4 complex (15 µM as dimer). The band shifts of the histone complexes were visualized by non-denaturing PAGE, followed by CBB staining. The reproducibility was confirmed by repeated experiments. (**D**) EMSA for p53ΔN binding to the nucleosomes, with or without p53BS. Increasing amounts of p53 (0, 0.2, 0.4, 0.6 and 0.8 µM) or p53ΔN (0.2, 0.4, 0.6 and 0.8 µM) were mixed with 0.1 µM of 193nuc (lanes 1–9) or 193nuc_BS (lanes 10–18). p53 or p53ΔN binding was detected by non-denaturing PAGE, followed by ethidium bromide staining. The reproducibility was confirmed by repeated experiments.

We next tested the histone-binding activity of p53 by a pull-down assay. As shown in Fig. 3A, we prepared two p53 truncation mutants, p53NTR and p53ΔN, which contained amino acid residues 1–93 and 94–393, respectively (Fig. 3A). Since the H3-H4 tetramer sits on the nucleosome dyad, and also binds to the entry/exit sites of the nucleosomal DNA (3, 4), we tested p53 binding to the H3–H4 tetramer. We prepared p53FL, p53NTR and p53ΔN as N-terminally GST-fused proteins, and performed the pull-down assay. As shown in Fig. 3B, the H3-H4 tetramer co-pelleted with p53FL or p53NTR, but not with p53ΔN (lanes 8–10). Consistently, the protein EMSA with un-tagged p53FL, p53NTR and p53ΔN revealed that p53FL and p53NTR efficiently bound to the H3-H4 tetramer, while p53ΔN did not (Fig. 3C). These results clearly indicated that p53 directly binds to the histone H3-H4 tetramer via its N-terminal amino acid region.

Finally, we tested whether the N-terminal region of p53 affects the nucleosome binding. To do so, we performed an EMSA with p53ΔN and the nucleosomes containing the 193 bp DNA. Although p53ΔN bound to the nucleosome, the bands corresponding to the specific p53-nucleosome complexes were barely detected (Fig. 3D). Therefore, the N-terminal region of p53 may contribute to its specific binding to the nucleosome, by binding to the H3-H4 tetramer.

The p53 N-terminal region contains TAD1 and TAD2 domains, which are required for p53 target gene induction (31). A histone acetyltransferase, p300, reportedly binds to the TAD1 and TAD2 domains of the p53 N-terminal region, and may induce the p53 target gene expression (32, 33). p53 forms multimers with its C-terminal region (34). Therefore, the p53 N-terminal region may function to connect p300 to histones in the nucleosome via p53 multimer formation. A well-characterized pioneer transcription factor, FoxA1, also interacts with the histone H3-H4 complex to induce an open chromatin configuration (35). The histone binding by p53 may be a common feature for a group of pioneer transcription factors.

## Abbreviations

CBB: Coomassie Brilliant Blue
CTD: C-terminus domain
CVs: column volumes
DBD: DNA-binding domain
EMSA: electrophoretic mobility shift assay
p53BS: p53 binding sequence
p53FL: p53 full-length
PAGE: polyacrylamide gel electrophoresis
PRR: proline-rich region
TAD1/2: Transcription activation domain ½
TD: tetramerization domain
